# Clot in the Blood Bag: A Case Report

**DOI:** 10.7759/cureus.69452

**Published:** 2024-09-15

**Authors:** Arthi Rajkumar, Soundharya Vetriveeran, Suresh Kumar Iyyapan, Hari Haran Annadurai, Sahayaraj James

**Affiliations:** 1 Transfusion Medicine, Saveetha Medical College and Hospital, Saveetha Institute of Medical and Technical Sciences, Chennai, IND

**Keywords:** blood bag, blood clot, capa, fish bone diagram, root cause analysis

## Abstract

Clot formation within a blood bag is a rare but significant issue, posing risks to the safety and quality of transfused blood products. We report the root cause analysis of a large clot found in a blood bag during routine component preparation. The analysis identified three potential contributing factors: improper vein selection leading to low flow rates, delays in tube stripping, and the use of a faulty blood collection monitor. These factors together facilitated the activation of coagulation, resulting in clot formation. To address these issues, corrective actions were implemented, including enhanced staff training on vein selection and phlebotomy techniques, timely and proper tube stripping procedures, and the replacement of faulty blood collection monitors with regularly calibrated equipment. Additionally, standard operating procedures (SOPs) were updated to incorporate these corrective measures. The implementation of these actions aims to prevent the recurrence of such incidents, ensuring the integrity of blood products and the safety of transfusion practices. This case highlights the importance of continuous monitoring and adherence to established protocols in blood collection and processing.

## Introduction

Blood transfusion is a critical procedure, relying on the safe collection, storage, and administration of blood products. The concept of blood bags, introduced in the early 20th century, marked a significant advancement in transfusion medicine. These bags are specially designed, flexible containers made primarily of polyvinyl chloride (PVC), facilitating the safe collection and storage of blood and its components [1]. Blood bags include various features, such as donor bags, tubing, and connectors, ensuring a sterile environment and ease of use during transfusion procedures.

Blood bags containing anticoagulants were first utilized by Rous and Turner in 1916 to prevent blood from clotting and to enhance the survival of red blood cells (RBCs) after transfusion [1]. Various anticoagulants have been used in combination, including citrate, phosphate, dextrose, and adenine [2]. Citrate chelates calcium and prevents clot formation. Phosphate maintains pH during storage. Dextrose and adenine help in adenosine triphosphate (ATP) production [3]. Proper mixing of the anticoagulant with the blood is crucial for optimal effectiveness. However, despite these safety measures, blood clots can still form in the bag. Smaller clots may go unnoticed, potentially causing flow issues during transfusion.

In this case report, we present a rare instance of a large clot discovered in a blood bag during routine component preparation. The incident was thoroughly investigated through root cause analysis (RCA), and corrective and preventive measures were implemented to prevent a recurrence. RCA is a systematic process for continuous quality improvement. This report aims to highlight the importance of vigilant blood product inspection and the need for stringent adherence to protocols.

## Case presentation

A 38-year-old male voluntary donor came to our blood bank to donate blood. The donor underwent screening and was found to be healthy, with no known history of coagulopathy or recent use of medications that could impact blood clotting. After meeting all the criteria outlined in the Drugs and Cosmetics Act, he was deemed eligible to donate. Informed consent was obtained from the donor before donation.

Phlebotomy was performed on the right upper limb under aseptic conditions. The blood collection was carried out using a Terumo triple blood bag, designed for the separation of whole blood into its components. Shortly after the procedure began, the blood collection monitor (BCM) triggered a low-flow alarm within the first minute of phlebotomy. Attempts to correct the flow by adjusting the needle position and increasing cuff pressure were unsuccessful, and the alarm continued. After four minutes, only 98 mL of blood had been collected, which was significantly less than the expected volume. Hence, it was decided to perform a second phlebotomy on the other limb.

Even after the second attempt at phlebotomy, the blood flow rate was only 5-6 mL per minute. The phlebotomy site was inspected by the nursing staff for any signs of hematoma or extravasation of blood, and the needle position was adjusted to increase the blood flow rate. The blood donor was advised to squeeze the sponge ball to raise the venous return, and the cuff pressure of the blood pressure apparatus was also raised to approximately 10 mmHg. Even after these interventions, there was no significant increase in blood flow rate.

**Figure 1:**
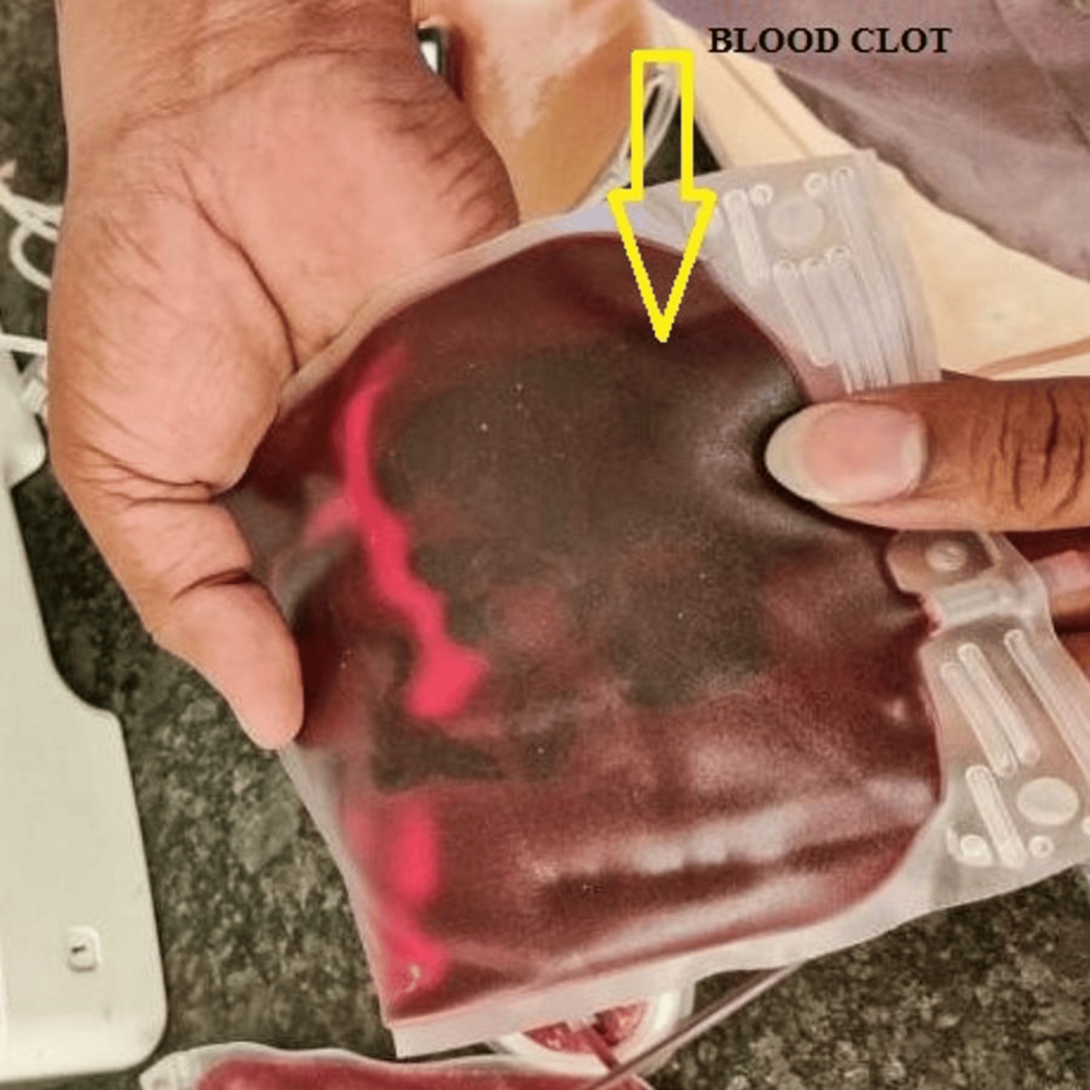
Blood bag with a large blood clot

A total of 350 mL of blood was collected, and the duration of the blood donation was around 12 minutes. During component separation, a large-sized blood clot measuring 6 cm × 7 cm (Figure 1) was identified in the blood bag by the technical staff, and it was informed to the blood bank medical officer.

**Figure 2:**
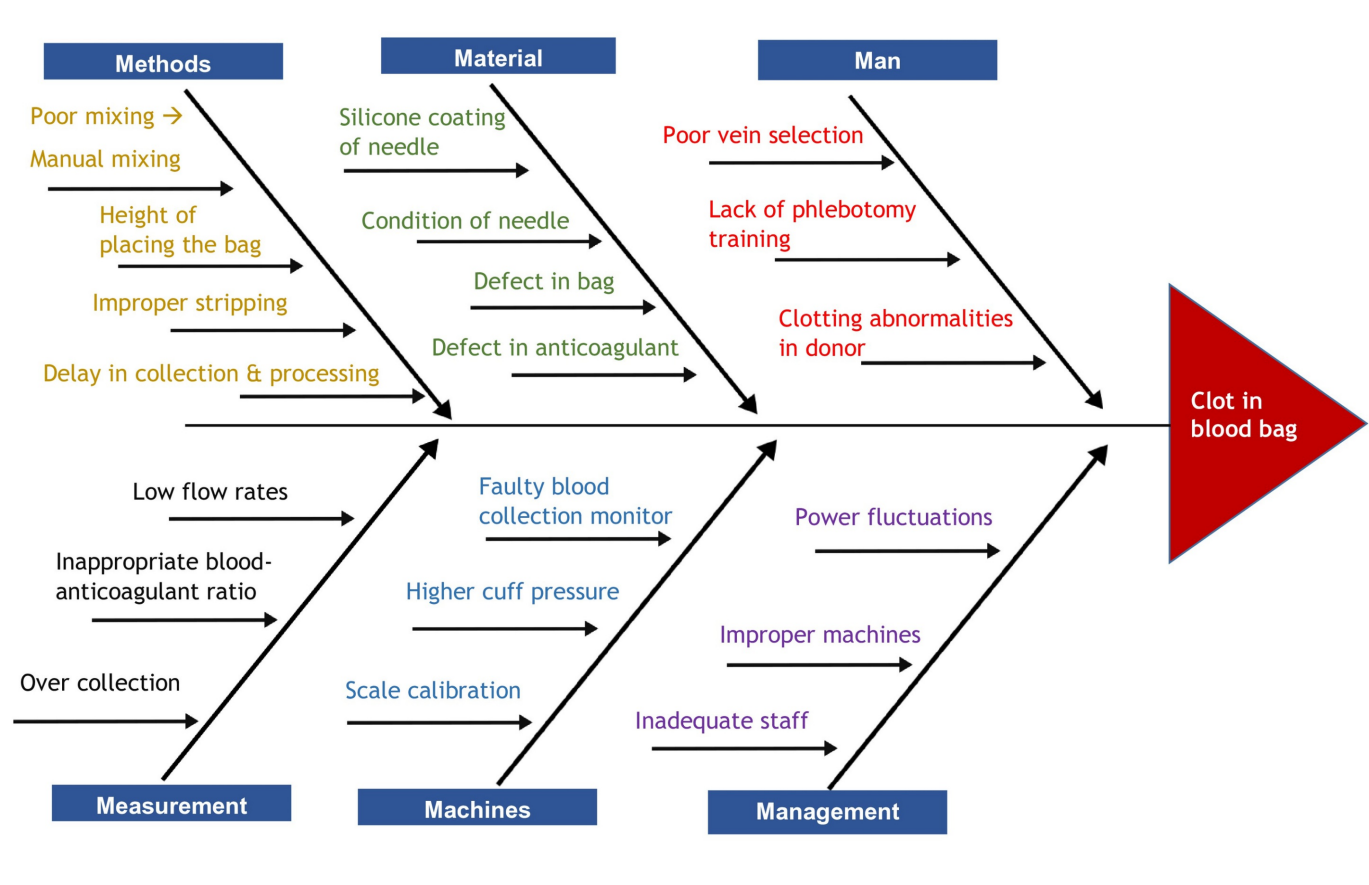
Fishbone analysis of blood clot formation in a blood bag

An RCA was conducted to identify all influencing and causal factors that contributed to this adverse event, following the fishbone analysis illustrated in Figure 2. This analysis demonstrated different factors related to personnel, machines, materials, and techniques that can influence clot formation [4].

The analysis identified three potential contributing factors: improper vein selection leading to low flow rates, delays in tube stripping, and the use of a faulty BCM. These factors together facilitated the activation of coagulation, resulting in clot formation. To address these issues, corrective actions were implemented, including enhanced staff training on vein selection and phlebotomy techniques, timely and proper tube stripping procedures, and the replacement of faulty BCMs with regularly calibrated equipment. Additionally, standard operating procedures (SOPs) were updated to incorporate these corrective measures.

## Discussion

In 1952, Dr. Carl Waldemar Walter of Harvard Medical School introduced the use of plastic bags for blood collection, replacing the fragile glass bottles that had been used previously. In 1981, the use of polyvinyl bags for collection, storage, and transfusion was legalized [1]. At present, di-(2-ethylhexyl) phthalate (DEHP) is added to PVC plastic to make the containers pliable. The needle used in blood bags is made from high-quality, thin-walled silicone, designed for smoothness to ensure a safe and comfortable blood donation experience during phlebotomy [2].

In 1943, J.F. Loutit and Patrick L. Mollison introduced an acid-citrate-dextrose (ACD) solution, which reduced the volume of anticoagulants needed while permitting longer-term storage of blood [1]. This advancement allowed for greater volumes of blood to be transfused, enhancing the efficiency of blood banking. The most commonly used anticoagulant is CPDA (citrate, phosphate, dextrose, adenine). Citrate chelates calcium, preventing clotting. Phosphate maintains pH. Dextrose and adenine help in ATP production. An adequate volume of blood is to be collected to maintain the ideal anticoagulant-to-blood ratio of 1:7 [4].

BCMs offer several benefits in standardizing the collection process. They help in the uniform mixing of the blood with the anticoagulant. BCM also helps to monitor flow rates and duration of blood collection. The monitor should be calibrated regularly for precise weight, blood collection volume, and the number of side-to-side movements per minute. It should also have a side-to-side movement rate of 16 cycles/minute [5]. An audio-visual alert for the intended blood collection volume should be included on the monitor. According to de Korte et al., the majority of BCMs are ineffective for mixing blood at low bleeding rates [6].

Whole blood should be collected into an approved container with a single, clean, non-traumatic venipuncture that allows rapid flow. The average draw time should vary between 5 and 10 minutes. However, the unit collected with a draw time beyond 10 and 12 minutes is not suitable for preparing platelet concentrates, and if the collection time exceeds 13 and 15 minutes, it is not suitable for preparing fresh frozen plasma (FFP) from 350 and 450 mL of blood bags, respectively [7]. After blood is collected, a tube stripper is used to remove blood from the blood bag tubing so that the blood is adequately mixed with anticoagulant.

Despite these advancements, blood clots can rarely be found in blood bags, as demonstrated in our case. Blood components such as platelets and plasma, which were separated from the same unit of the bag with clot, must be identified and quarantined as per the Association for the Advancement of Blood & Biotherapies (AABB) guidelines [8]. An RCA commences with the collection of information from technicians and nursing staff involved with this donor unit. The anticoagulant present in the blood bag was within the expiry date. The right amount of pressure was applied above the venipuncture site using a blood pressure cuff (between 40 and 60 mmHg). The medication list of the donor was checked, and the clotting abnormality was ruled out. Despite all such attempts, there was a prolonged blood collection time of approximately 12 minutes that led to clot formation.

Three causative factors were identified for the formation of the large clot. The first was poor vein selection, leading to a low flow rate, defined as less than 10 mL every 30 seconds. Additionally, as per the bag design, the absence of an anticoagulant coating on the initial 90-cm tube connected to the bag may have contributed to the activation of coagulation. To prevent wasting the bag and the valuable resource, we frequently carry out the collection even in the presence of a low flow alert. As a corrective measure, staff have been trained to identify suitable veins and apply proper phlebotomy techniques. They have also been instructed to monitor the flow rate on the collection monitor in the event of an alarm and to halt the collection if the issue is not resolved within two minutes. The latest blood collection technology, which includes a safety feature that triggers an automatic clamp if the flow rate remains below 20 mL per minute for more than two minutes, can also be utilized.

The second factor identified was improper stripping techniques. After collection, the needle is sealed and cut, and the blood remaining in the tubing is pushed into the bag using a tube stripper to mix with the anticoagulant. A delay in stripping may have contributed to clot formation in the tubing, which could have been transferred into the bag during the stripping process. This delay was often due to insufficient staffing during busy periods, where multitasking led to the postponement of proper stripping procedures. To address this issue, staff were educated on the importance of timely and thorough stripping of the tubes. Additionally, extra staff were requested during peak times to ensure that all procedures could be carried out promptly and effectively.

**Figure 3:**
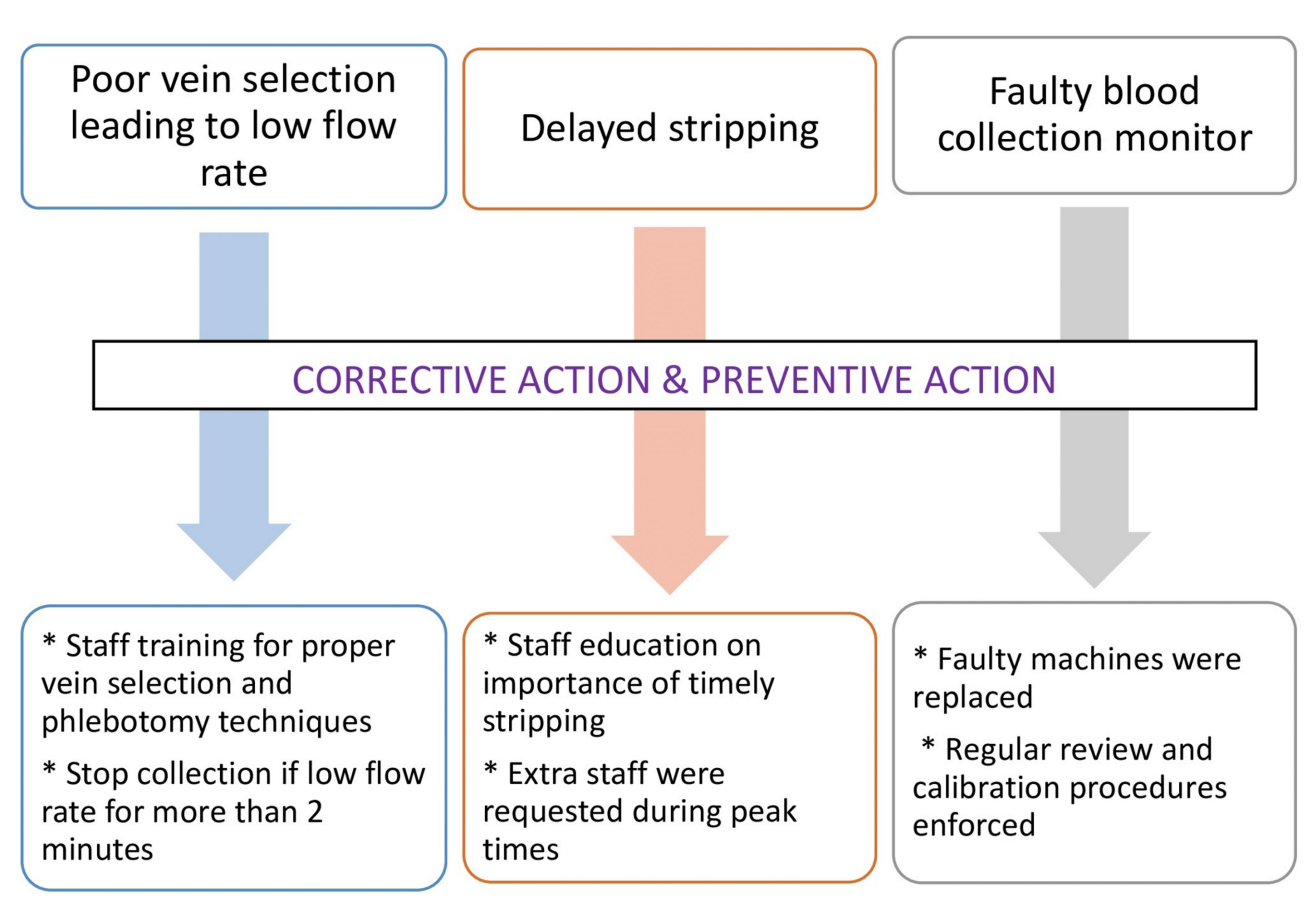
Corrective and preventive measures implemented

The third issue identified was a faulty BCM. The BCM had not been recently calibrated, and power fluctuations during collection caused it to stop, potentially leading to inaccurate volume measurement. To address this, the faulty machines were replaced with properly calibrated ones, and regular review and calibration procedures were enforced. Our SOPs were updated to incorporate these corrections. Additionally, we emphasized to our technical team the critical importance of adhering to the SOPs during whole blood collection and component preparation. Figure 3 depicts a summary of the corrective and preventive measures implemented.

## Conclusions

The RCA of the clot found in the blood bag identified several key factors contributing to the issue: improper vein selection leading to low flow rates, delays in tube stripping, and the use of faulty blood collection equipment. These factors collectively resulted in the activation of coagulation and clot formation within the blood bag.

As a result of this analysis, corrective actions have been implemented, including enhanced staff training on vein selection and phlebotomy techniques, timely and proper tube stripping, and the use of calibrated BCMs. Additionally, SOPs have been updated to reflect these changes, ensuring that all processes are aligned with best practices. This comprehensive approach will help prevent similar incidents in the future, safeguarding the quality of blood products and maintaining the safety and efficacy of transfusion procedures.
